# RNA cytosine methyltransferase Nsun3 regulates embryonic stem cell differentiation by promoting mitochondrial activity

**DOI:** 10.1007/s00018-017-2700-0

**Published:** 2017-11-04

**Authors:** Lukas Trixl, Thomas Amort, Alexandra Wille, Manuela Zinni, Susanne Ebner, Clara Hechenberger, Felix Eichin, Hanna Gabriel, Ines Schoberleitner, Anming Huang, Paolo Piatti, Roxana Nat, Jakob Troppmair, Alexandra Lusser

**Affiliations:** 1 Division of Molecular Biology, Biocenter, Medical University of Innsbruck, Innrain 80-82, 6020 Innsbruck, Austria; 2 Daniel Swarovski Research Laboratory, Department of Visceral, Transplant, and Thoracic Surgery, Medical University of Innsbruck, 6020 Innsbruck, Austria; 3 Zymo Research Corp., Irvine, CA USA; 4 Institute for Neuroscience, Medical University of Innsbruck, 6020 Innsbruck, Austria

**Keywords:** tRNA modification, 5-Methylcytosine, Mitochondria, Bisulfite sequencing, Neuroectoderm, Self-renewal, Epitranscriptome

## Abstract

Chemical modifications of RNA have been attracting increasing interest because of their impact on RNA fate and function. Therefore, the characterization of enzymes catalyzing such modifications is of great importance. The RNA cytosine methyltransferase NSUN3 was recently shown to generate 5-methylcytosine in the anticodon loop of mitochondrial tRNA^Met^. Further oxidation of this position is required for normal mitochondrial translation and function in human somatic cells. Because embryonic stem cells (ESCs) are less dependent on oxidative phosphorylation than somatic cells, we examined the effects of catalytic inactivation of Nsun3 on self-renewal and differentiation potential of murine ESCs. We demonstrate that *Nsun3*-mutant cells show strongly reduced mt-tRNA^Met^ methylation and formylation as well as reduced mitochondrial translation and respiration. Despite the lower dependence of ESCs on mitochondrial activity, proliferation of mutant cells was reduced, while pluripotency marker gene expression was not affected. By contrast, ESC differentiation was skewed towards the meso- and endoderm lineages at the expense of neuroectoderm. *Wnt3* was overexpressed in early differentiating mutant embryoid bodies and in ESCs, suggesting that impaired mitochondrial function disturbs normal differentiation programs by interfering with cellular signalling pathways. Interestingly, basal levels of reactive oxygen species (ROS) were not altered in ESCs, but Nsun3 inactivation attenuated induction of mitochondrial ROS upon stress, which may affect gene expression programs upon differentiation. Our findings not only characterize Nsun3 as an important regulator of stem cell fate but also provide a model system to study the still incompletely understood interplay of mitochondrial function with stem cell pluripotency and differentiation.

**Electronic supplementary material:**

The online version of this article (10.1007/s00018-017-2700-0) contains supplementary material, which is available to authorized users.

## Introduction

RNA modifications have been shown to play important roles in the function and metabolism of all types of RNA in eukaryotes and in bacteria. Particularly, tRNAs are extensively modified, which is reflected in the fact that they carry approximately 90 out of the ~ 150 known RNA modifications [1]. The pattern of tRNA modification ranges from highly conserved types of modifications at highly conserved nucleosides to modifications that are unique to specific residues of (a) specific tRNA (group) [2]. Consistent with the great diversity of modifications, they are involved in a wide range of functions. tRNA modifications are required for proper tRNA folding, they can affect aminoacylation, regulatory tRNA cleavage, as well as interaction with and decoding of the mRNA during translation [3–6]. Stress-induced changes in modification patterns regulate tRNA cleavage, which, in turn, results in downregulation of protein biosynthesis to allow for the repair of cell damage or apoptosis and might, therefore, be important for the modulation of cancer cell metabolism [7]. tRNA modification defects have also been associated with mitochondrial disease [4, 8]. For instance, loss of the taurine modification at position U34 in the anticodon loop of human mt-tRNA^Leu(UUR)^ was detected in patients suffering from MELAS (mitochondrial encephalomyopathy, lactic acidosis, and stroke-like episodes) syndrome [4]. 5-Taurinomethyluridine was shown to stabilize wobble pairing with codons that end in a G [9]. Another mitochondrial anticodon loop modification is 5-formylcytidine (f5C) at position C34 of mt-tRNA^Met^, which stabilizes base-pairing with A to enable decoding of the non-universal mitochondrial AUA in addition to the universal AUG codon [10, 11]. Recently, four studies have identified the enzymes that are responsible for the generation of f5C in mt-tRNA^Met^. It was shown that the RNA cytosine methyltransferase (RCMT) NSUN3 targets C34 for carbon 5 methylation in human cells [12–14], while the alpha-ketoglutarate and Fe(II)-dependent dioxygenase ALKBH1/ABH1 further oxidizes 5-methylcytidine (m5C) to f5C [14, 15]. *NSUN3* deletion abolished f5C in tRNA^Met^ of human dermal fibroblasts, HeLa, and HEK293 cells and led to impaired mitochondrial translation efficiency presumably by interfering with efficient decoding of the AUA codons in mitochondrially encoded transcripts of electron transport chain components [12–14]. Functional inactivation of NSUN3 as well as point mutations found in patients that occur in the vicinity of C34 on mt-tRNA^Met^ and affect NSUN3-mediated methylation resulted in mitochondrial disease [12, 13].

Adult somatic cells rely heavily on oxidative phosphorylation (OXPHOS) in mitochondria to meet their energy demands. Therefore, defects in the electron transport chain typically have severe consequences for cell metabolism. By contrast, embryonic stem cells (ESCs) predominantly utilize anaerobic glycolysis, and it has been demonstrated that their mitochondria show reduced respiration, they have globular shape and perinuclear localization [16, 17]. Reprogramming of somatic cells to pluripotent stem cells is accompanied by morphological changes of mitochondria and a downregulation of electron transport chain complex I and II subunits [18]. Nevertheless, although mitochondria in stem cells may not be essential for ATP production, they appear to support stemness by enforcement of alternative pathways, such as threonine catabolism in murine but not human ESCs or by channelling intermediates from the tricarboxylic acid cycle for anabolic pathways [19]. Differentiation of ESCs, on the other hand, is accompanied by a shift from glycolytic to oxidative metabolism reflected in a gain in mitochondrial mass, upregulation of mitochondrial enzymes and downregulation of glycolytic enzymes, increased oxygen consumption, and lower lactate production. ESC differentiation is also affected by mitochondrial reactive oxygen species (ROS), although the exact mechanisms in ESCs are not well understood [20].

Given the impact of C34 modification in mt-tRNA^Met^ on mitochondrial translation of electron transport chain components in human somatic cells [12, 13], we examined if C34 modification also plays a critical role in mouse ESCs despite their favouring anaerobic glycolysis over OXPHOS. We catalytically inactivated the C34 methyltransferase Nsun3 in mouse ESCs by CRISPR/Cas9 and examined the functional consequences on ESC self-renewal, stemness, energy metabolism, and differentiation potential.

## Materials and methods

### Embryonic stem cell culture and differentiation

Mouse embryonic stem cells (129/Sv) were cultured in ESC medium (LIF+2i) (DMEM high glucose with GlutaMAX-1 [Gibco], 20% FBS [Gibco], 1 × non-essential amino acid mix [Gibco], 0.05 mM β-mercaptoethanol, 10 µg/ml LIF [Sigma], 3 µM CHIR99021, 1 µM PD0325901 [both Axon Medchem]) in gelatine-coated culture dishes at 37 °C and 5% CO_2_. Induction of embryoid body (EB) formation and EB outgrowth were performed as previously described [21]. Differentiation of ESCs into the ectodermal lineage was performed as described previously [22]. In brief, ESCs were cultured in N2B27 supplemented serum-free medium, containing 10 µg/mL LIF, 3 µM CHIR99021, and 1 µM PD0325901 for 24 h in 25 cm^2^ flasks before passaging to 6-well plates in the same medium containing only 0.4 µM PD0325901 for 2 days. After that, cells were incubated with 1 µM of LDN193189 (BMP antagonist; Sigma) for additional 4 days.

### Catalytic inactivation of Nsun3 in mouse ESCs

To generate an ESC cell line expressing catalytically inactive Nsun3, the CRISPR/Cas9 method was used [23]. A double-stranded oligo containing the sgRNA sequence targeting the catalytically important T_264_C_265_ motif encoded in exon 6 of mouse *Nsun3* (NC_000082.6) was cloned into the vector pX458 [23], which encodes GFP in addition to the Cas9 nuclease. The recombinant plasmid was transfected into ESCs using Lipofectamine 2000 (Invitrogen) according to the manufacturer’s instructions and cultured for 24 h. Cells were then trypsinized and subjected to FACS sorting of single GFP^+^ cells into 96-well plates containing 200 µl of a 1:1 mixture of preconditioned and fresh ESC medium. After about 6 days, ES cell colonies were visible. Several colonies were expanded and screened for indel mutations in *Nsun3* exon 6. To this end, PCR (NSUN3fw 5′ AGCTTTGCCCTTTTCCGGAA and NSUN3rev 5′ CGTGCTGTGATGATCCCCAA) was performed to amplify the region around the targeting site using the Terra PCR Direct Polymerase Mix (Clontech). Genomic DNA extraction and PCR were performed according to the manufacturer’s instructions. PCR products were subcloned into pGEM-T Easy vector (Promega) and sequenced.

### Nsun3-GFP construct generation, transfection, and Nsun3 localization

The pX458 vector [23] was digested with *AgeI* and *BsrG*I restriction enzymes. The *Nsun3* ORF was amplified from mouse cDNA using the following primers: NSUN3_fw: 5′ TCA CTT TTT TTC AGG TTG GAG CCA CCA TGC TGA CTC GGC TGA AAG 3′ and NSUN3_rev: 5′ CCT TGC TCA CTG GCT TAC AAA ATT CGC C 3′. The GFP ORF was amplified from a pMT_EGFP plasmid [24] with the following primers: GFP_fw: 5′ TTG TAA GCC AGT GAG CAA GGG CGA GGA G 3′ and GFP_rev: 5′ AGC TCT AGT TAG AAT TCC TTT CAC TTG TAC AGC TCG TCC 3′. Both fragments and the vector were fused using the NeBuilder (NEB) system to generate a construct for the expression of Nsun3 with a C-terminal EGFP tag. The plasmid was transfected into mouse ESCs using Lipofectamine 2000 (Thermo Fisher Scientific) following the standard methods. 24 h post-transfection, mESCs were seeded into 8-well chambered Nunc Lab-Tek coverglasses and stained using 100 nM MitoTracker^®^ Red CM-H_2_XRos (Invitrogen) in serum-free DMEM for 30 min at 37 °C in a 5% CO_2_ environment. Afterwards, medium was replaced by complete ESC medium and live cell images were taken using a Leica SP5 confocal microscope. For Alkbh1 localization, mESCs were transfected with 4 µg of the pX-Nsun3-GFP plasmid using lipofectamine. 24 h post-transfection, cells were detached by Accutase^®^ (Sigma) to obtain single cells and subsequently allowed to settle down on cover slips. Fixing, antibody incubation and washing was performed exactly as described before [21]. Antibodies used: anti-GFP (1:10,000; Thermo Fisher, A-11122) and anti-ALKBH1 (1:100; Abnova, H00008846-B01P). DAPI was used to visualize DNA. Cell images were taken as described above. Images were processed using ImageJ and Affinity Photo (Serif) software.

### ESC proliferation measurements

Cell proliferation was determined using the CyQuant^®^ Cell Proliferation Assay Kit (ThermoFisher) according to the manufacturer’s instructions. 3000 cells were seeded in five technical replicates in 96-well plates in ESC medium. Every 24 h, cells were harvested, washed in PBS, and frozen at − 70 °C. At the end, all cell samples were resuspended in 200 µl each of CyQuant^®^ GR dye/cell-lysis buffer and fluorescence was measured using an FLUOstar^®^ Omega microplate reader at 480 nm setting. Fluorescence values were converted to cell numbers using the linear equation of a standard curve that was generated by measuring serial dilutions of a known number of cells.

### RNA extraction, RT-qPCR, and strand-specific Northern blotting

RNA extraction and reverse transcription real-time PCR (RT-qPCR) were performed essentially as described previously [21] on cDNA obtained from at least three independent biological replicates. Primer sequences are available upon request. Statistical significance of differences between mutant and wild-type samples was determined using multiple unpaired *t* test with Holm–Sidak correction for multiple testing (Graphpad Prism 7.0). Expression levels of mitochondrially encoded transcripts were analyzed by separating 5 µg total RNA on 1.2% agarose/1.1% formaldehyde gels, blotting onto Hybond-N membrane (GE Healthcare) and hybridizing with DIG-labelled strand-specific RNA probes in “High-SDS” solution (7% SDS, 50 mM Na-phosphate buffer, pH 7.0, 50% deionized formamide, 5 × SSC, 2% Roche blocking solution, 0.1% Na-lauroylsarcosine). Since both strands of the mitochondrial genome are transcribed, strand-specific ssRNA probes were generated by in vitro transcription of PCR amplified fragments of mitochondrial transcripts using the MEGAScript T7 in vitro transcription kit (Thermo Fisher Scientific) in combination with the DIG RNA labelling mix (Roche) according to the manufacturer’s instructions. After hybridization, northern blots were subjected to stringent washes in 0.1 × SSC/0.1% SDS at 68 °C and finally to DIG detection following the Roche protocol. Chemiluminescent signal was captured using a Fusion SL3500 WL instrument (Vilber) or X-ray film. Signal intensities were quantified using the Image Studio Lite (v5.2) software. Statistical significance of differences was calculated by multiple unpaired *t* test analysis in Graphpad Prism 7 (*p < 0.05).

### Reductive treatment of RNA and RNA bisulfite sequencing

To reduce 5-formylcytosine to 5-hydroxmethlycytosine, RNA was treated with sodium borohydride following a previously described method [25]. Briefly, 1.5 µg of total RNA in 15 µl were mixed with 5 µl of a freshly prepared 1 M NaBH_4_ solution (in water) and incubated for 30 min at room temperature in the dark. The reaction was stopped by addition of 10 µl 750 mM sodium acetate (pH 5) and kept at room temperature until no further gas was released. Samples were then subjected to PCR-mediated bisulfite sequencing exactly as described before [26]. cDNA synthesis was performed with an mt-tRNA^Met^-specific stem loop primer (5′ CTCAACTGGTGTCGTGGAGTCGGCAAT TCAGTTGAGTGGTTAAACCAAC); mt-tRNA^Met^ was then amplified with primers DtRNAm fw 5′ AAGGTTAGTTAATTAAGTTATT and UniSL rev 5′ CACGACACCAGTTGA, subcloned into pGEM-T vector (Promega), and inserts were subjected to Sanger sequencing.

### Analysis of mitochondrial translation by metabolic labelling

Pulse labelling of mitochondrial proteins was performed as described before [27], with the following changes: 9.5 × 10^5^ wild-type and *Nsun3*-mutant ES cells per well were seeded into a gelatine-coated 6-well plate and cultivated for 24 h. Cells were washed once with PBS followed by one wash with labelling medium (ESC medium without cysteine and methionine) and a 30 min incubation in 1.8 ml labelling medium. Then, 200 µl of emetine (1 mg/ml) were added and incubated at 37 °C for 40 min to stop cytoplasmic translation. To label mitochondrially translated products, 40 µl of l-[^35^S]-methionine (10 mCi/ml; Hartman Analytic) were added and the cells were incubated for 1 h. Cells were washed in standard ESC medium and incubated another 10 min at 37 °C. Cells were then harvested by trypsinizing, washed once with ice-cold PBS, and extracted with ice-cold RIPA buffer containing protease-inhibitors. Proteins were fractionated by gel electrophoresis in 16% Tricine gels (Invitrogen) and stained with Coomassie brilliant blue, and radioactive signals were visualized by phosphoimaging. Signal intensities were quantified using the Image Studio Lite (v5.2) software. Statistical significance of differences was calculated by multiple unpaired *t* test analysis in Graphpad Prism 7 (*p < 0.05).

### Real-time PCR-based determination of mitochondrial DNA content

DNA was extracted from wild-type and *Nsun3* ^*cat/cat*^ cells using standard protocols. 100 ng of DNA was subjected to real-time PCR to amplify a specific region in nuclear DNA (apoBfw 5′ CGTGGGCTCCAGCATTCTA and apoBrev 5′ TCACCAGTCATTTCTGCCTTTG) and two regions in the mitochondrial genome (*Mito1_ND5*: ND5fw 5′AATAGTGACGCTAGGAATAA and ND5rev 5′GATGTCTTGTTCGTCTGCCA; *Mito2_ Rnr2*: Rnr2fw 5′AGGGATAACAGCGCAATCCT and Rnr2rev 5′AGGGATAACAGCGCAATCCT). Mitochondrial amplification signals were normalized against the nuclear signal and expressed relative to wild-type ESCs. Mean values ± SEM of three experiments are shown and statistical significance was calculated by unpaired *t* test (*p < 0.05).

### Respiration and glycolysis measurements

Oxygen consumption rate (OCR) and extracellular acidification rate (ECAR) were determined with a Seahorse XFe96 Analyzer (Seahorse Bioscience). To this end, 40,000 wild-type or mutant ESCs were seeded into 7–8 wells of a gelatine-coated 96-well analyzer plate (Seahorse Bioscience) and incubated overnight under standard conditions. For determination of OCR, oligomycin (2 μM), carbonyl cyanide 4-(trifluoromethoxy) phenylhydrazone (FCCP) (0.6 µM), and rotenone/antimycin A (0.5 μM) were injected according to the XF Cell Mito Stress Test Kit (Seahorse Bioscience). Glycolytic function was determined using the XF Glycolysis Stress Test Kit (Seahorse Bioscience). Glucose (10 mM), oligomycin (2 µM), and 2-deoxy-glucose (2-DG) (50 mM) were injected according to the manufacturer’s protocol. The obtained OCR (respiration) and ECAR (glycolysis) data were normalized against cell numbers in each well and analyzed using the Wave software as well as the XF Report Generator (Seahorse Bioscience). Cell numbers were determined by Hoechst 33342 (Thermo Scientific) staining combined with fluorescence measurement using Infinite F200 PRO instrument (Tecan).

### ROS and mitochondrial membrane potential measurements

Mitochondrial ROS were imaged by fluorescence microscopy after staining the cells with 100 nm MitoTracker Red CM-H_2_XROS (Thermo Fisher Scientific) in serum-free DMEM medium. For this purpose, 50,000–80,000 cells/well were seeded in the 8-well Nunc Lab-Tek II Chamber Slide System (Thermo Fisher Scientific). For ROS measurements in cells undergoing differentiation, varying starting cell numbers were seeded into the individual wells depending on the duration of culture: 50,000–80,000 (15 h), 25,000–35,000 (28 h), and 10,000–15,000 cells/well (48 h) in ESC medium with LIF and 2i. After 16 h cells were either left untreated or differentiation was induced by medium without LIF and 2i. ROS staining was performed for 30 min at 37 °C without stress or after stressing the cells by incubation in 1 mM H_2_O_2_ in ESC medium for 30 min at 37 °C. Digital images were taken using an Olympus IX-70 inverted microscope (Olympus America) with an Olympus 40 × water immersion objective (numerical aperture 0.8) and an Olympus U-RFL-T mercury-vapor lamp. Image acquisition was performed with a Kappa ACC1 camera and Kappa ImageBase software (Kappa Opto-electronics). For MitoTracker Red CM-H_2_XROS, a 568-nm filter was used. Gray values were measured using the Scion Image software for Windows. For every experimental condition, gray values from 100 to 120 cells were averaged.

Total cellular ROS levels were determined by staining with 2′,7′-dichlorofluorescin-diacetate (DCF-DA) (Sigma-Aldrich) after stress application as described above. Cells were loaded with 10 μM DCF-DA and incubated for 15 min at 37 °C before FACS measurement. Quantitative analysis was done using the CellQuest software for FACSCalibur (BD Biosciences).

To analyze mitochondrial membrane potential, control or H_2_O_2_-stressed cells were incubated for 15 min at 37 °C with 25 nM tetramethylrhodamine methyl ester (TMRM, Invitrogen). TMRM fluorescence was detected by FACS. In control experiments, dissipation of membrane potential was observed after addition of 5 µM carbonyl cyanide 4-(trifluoromethoxy)phenylhydrazone (FCCP, Sigma-Aldrich). Values were expressed as geometric means after correction for background TMRM fluorescence measured in uncoupler (FCCP)-treated cells.

Statistical analyses were performed in Graphpad Prism 7 using one-way ANOVA with Bonferroni correction.

## Results

### Generation of ESCs with catalytically inactive Nsun3

**Fig. 1:**
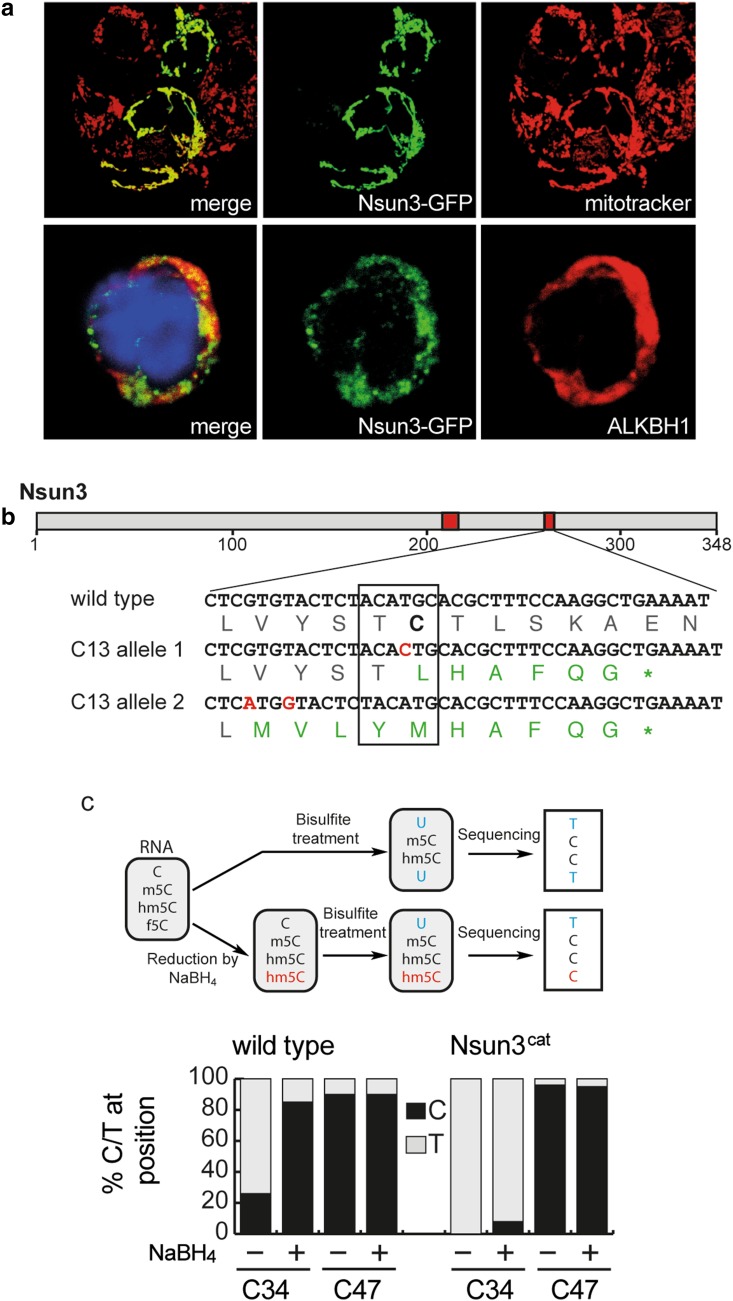
Nsun3 localization and catalytic inactivation of *Nsun3* in embryonic stem cells. **a** Nsun3 and Alkbh1 colocalize in mitochondria of murine ESCs. Cells were transiently transfected with an *Nsun3*-*GFP* construct and co-labelled with mitotracker (upper panels) or stained with antibodies against GFP and Alkbh1 (lower panels). DNA was visualized by DAPI staining. **b** Schematic representation of Nsun3 protein domains. Red boxes, catalytically important areas around PC and TC motifs. Sequencing results of mutated clone shows amino acid changes in both alleles around the catalytically important T_263_C_264_ motif (boxed) and a premature stop codon. **c** RNA from *Nsun3*-mutant and wild-type cells was subjected to bisulfite sequencing and NaBH_4_-bisulfite sequencing to determine the modification levels of C34 and C47 as illustrated in the schematic representation. *Nsun3* ^*cat/cat*^ cells lose methylation and formylation at C34 but not methylation at C47 of mt-tRNA^Met^. RNA was analyzed by bisulfite sequencing with (+) or without (−) preceding NaBH_4_ treatment to reduce 5-formylcytosine to 5-hydroxymethylcytosine

To study the role of RNA methyltransferase activity of Nsun3 in ESCs and ESC differentiation, we first examined if Nsun3 localizes to mitochondria in ESCs by transiently expressing GFP-tagged Nsun3. Co-labelling with mitotracker, indeed, revealed perfect co-localization of Nsun3 with mitochondria (Fig. 1a). We then set out to generate a catalytically inactive mutant of Nsun3. RCMTs of the NSUN type use two conserved cysteines in their catalytic domain for methyl group transfer to cytosine. The cysteine located within a conserved threonine–cysteine (TC) motif (T_264_C_265_ in murine Nsun3) is assumed to form a covalent adduct with carbon 6 of cytosine initiating the transfer of a methyl group from SAM to C5, while the second cysteine (C_214_ in Nsun3) located within a proline-cysteine (PC) motif presumably acts as a base to resolve the covalent bond and release the enzyme [28–30]. We employed CRISPR/Cas9 technology to mutate the TC motif in Nsun3 to inactivate its catalytic activity. This approach led to the isolation of an ESC clone (*Nsun3* ^*cat/cat*^), in which both alleles of the *Nsun3* gene were targeted by the guide RNA and which is predicted to give rise to a protein with mutated TC motif and, due to the insertion of an additional nucleotide in both alleles, to a premature stop codon, which results in truncation of 70 amino acids from the C-terminus (Fig. 1b). Since NSUN3 was recently found to methylate mitochondrial tRNA^Met^ at C34 in the anticodon loop [12–14], which is then further oxidized to 5-formylcytosine by ALKBH1/ABH1 [14, 15], we analyzed the methylation status of mt-tRNA^Met^ in mouse ESCs. To this end, we subjected isolated RNA to bisulfite sequencing [26] and found that 26% of clones showed non-conversion of C to U in wild-type cells indicating the presence of m5C at C34. By contrast, C34 was completely converted to uracil in *Nsun3* ^*cat/cat*^ cells (Fig. 1c). Since bisulfite treatment does not distinguish between formylated and non-modified cytosine, we subjected the RNA to reduction by sodium borohydride to convert 5-formylcytosine (f5C) to 5-hydroxymethylcytosine (hm5C), which is resistant to bisulfite-mediated deamination [25] (see schematic in Fig. 1c). We found that 85% of C34 in mt-tRNA^Met^ displayed a C instead of a U after the treatment in wild-type ESCs, while this was the case for only 8% in *Nsun3* ^*cat/cat*^ cells (Fig. 1c). Thus, like in human cells, C34 of mt-tRNA^Met^ of mouse ESCs is predominantly formylated and to a lower degree present in a methylated or unmodified state. However, since neither bisulfite sequencing nor NaBH_4_-bisulfite sequencing is able to distinguish between hm5C and m5C or f5C and hm5C, respectively (Fig. 1c, schematic), a small portion of what we denote as m5C34 mt-tRNA^Met^ may correspond to hm5C34, which is generated as an intermediate on the pathway to formylation [15].

Formylation of C34 is carried out by ALKBH1 [14, 15], and like NSUN3, ALKBH1 localized to mitochondria in HEK293 cells [14]. Consistent with this, co-staining of Alkbh1 with GFP-tagged Nsun3 in mouse ESCs revealed a clear although not perfect overlap suggesting mitochondrial localization of Alkbh1 (Fig. 1a). This result contrasts the finding by Ougland et al. for human ESCs, where ALKBH1 was shown to be predominantly present in the nuclei but not in mitochondria [31, 32]. It is possible that mouse and human ESCs are different with respect to ALKBH1 localization. However, we consider it likely that a small portion of Alkbh1 is also present in nuclei and/or in the cytoplasm of mouse ESCs or of other cell types, since it was recently found to be involved in demethylation of cytoplasmic m1A-modified tRNAs and in the oxidation of m5C34 of cytoplasmic tRNA^Leu^ in HEK293 and HeLa cells [15, 33].

Besides m5C34, we detected a previously unknown methylation at C47 in the variable loop of mt-tRNA^Met^ that is not conserved in human mt-tRNA^Met^. The extent of C47 methylation was virtually complete not only in ESCs but also in differentiating ESCs, adult brain tissue and in MEFs (Fig. 1c, Supplementary Fig. S1). Consistent with Nsun3 specifically targeting C34, C47 methylation did not change in the *Nsun3*-mutant ESCs (Fig. 1c). Thus, while wild-type ESCs possess methylated and formylated C34 in mt-tRNA^Met^, *Nsun3*-mutant cells exhibit loss of C34 methylation and strong reduction of formylation indicating that we have generated a cell line expressing a catalytically inactive Nsun3 protein (hereafter termed Nsun3^cat^).

### Nsun3^cat^ affects proliferation but not pluripotency marker expression in ESCs

**Fig. 2:**
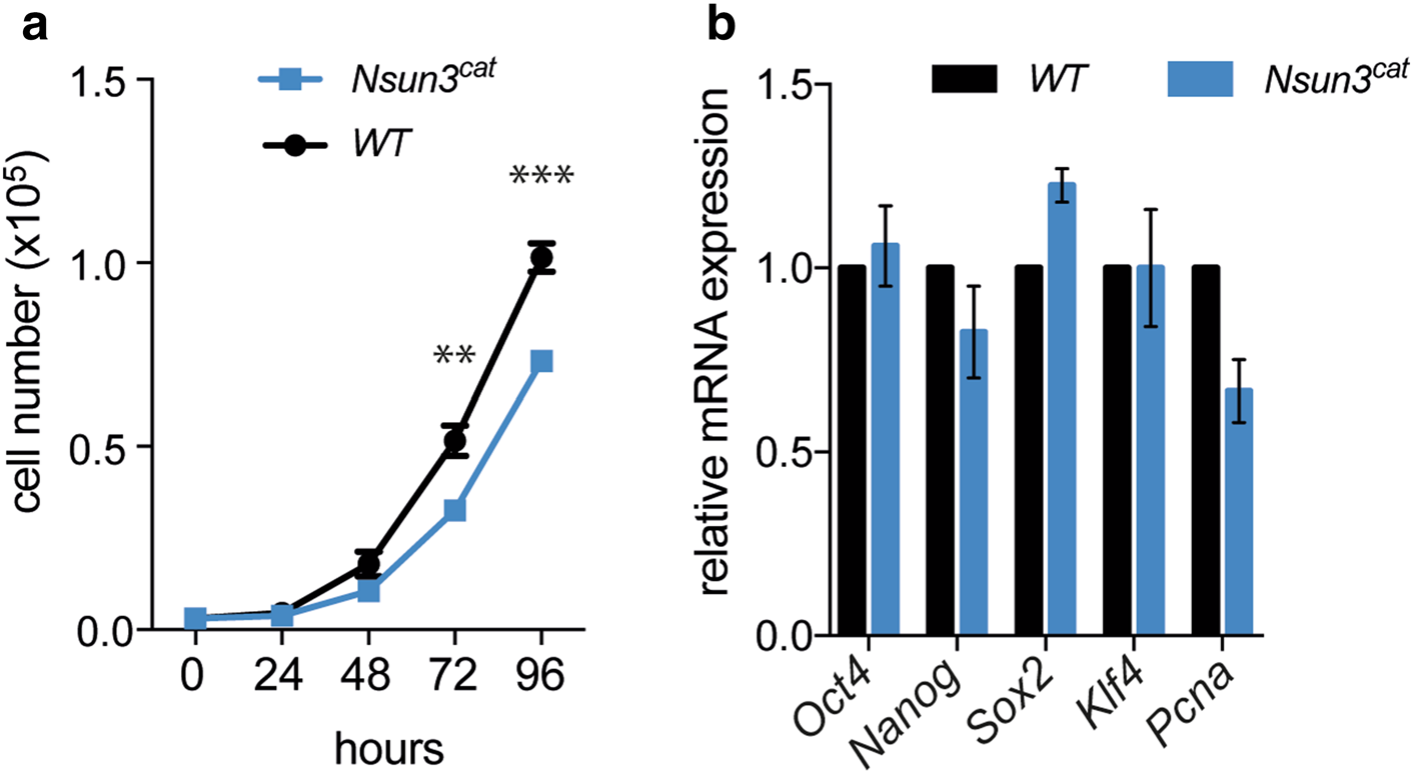
Cell proliferation but not pluripotency marker expression is altered in *Nsun3* ^*cat/cat*^ ESCs. **a** Wild-type and *Nsun3*-mutant cells were seeded into 96-well plates and cell numbers were determined using the CyQuant^®^ fluorescence assay at the indicated time points. Mean ± SEM of five technical replicates are shown. Statistically significant differences were found for 72 h (**p = 0.007) and 96 h (***p = 0.0004) values by unpaired *t* test. **b** RT-qPCR was performed to determine the expression levels of pluripotency markers in *Nsun3* ^*cat/cat*^ relative to wild-type cells. Mean ± SEM of three experiments are shown. Statistical significance was determined by multiple unpaired *t* test with Holm–Sidak correction (*p < 0.05)

To investigate potential effects on ESC physiology, we analyzed the proliferation rate of wild-type versus *Nsun3* ^*cat/cat*^ cells over 96 h using the CyQuant^®^ cell proliferation assay. Cell numbers of *Nsun3* ^*cat/cat*^ ESCs showed a significant reduction after 3 and 4 days in culture compared to wild-type cells (Fig. 2a). Similar results were obtained by counting the cells in a hemocytometer (data not shown). This was supported by a trend to lower transcript levels of the replication marker proliferating-cell-nuclear-antigen (Pcna, *p* = 0.058; Fig. 2b). Despite the slightly slower proliferation rate, FACS analyses revealed no changes in the cell cycle profile (data not shown). We next examined, if *Nsun3* mutation affects stem cell features of ESCs. Analysis of expression levels of the major pluripotency markers *Oct4*, *Nanog*, *Sox2,* and *Klf4,* however, showed no significant differences between wild-type and mutant cells (Fig. 2b), while the early differentiation markers *Bra*, *Gata4*, *Gata6*, *Sox1,* and *Pax6* were not expressed in both wild-type and mutant cells (data not shown). Thus, we conclude that the catalytic activity of Nsun3 is not required for maintaining the undifferentiated state of ESCs, but its loss negatively affects cell proliferation.

### Catalytically inactive Nsun3 compromises mitochondrial activity

**Fig. 3:**
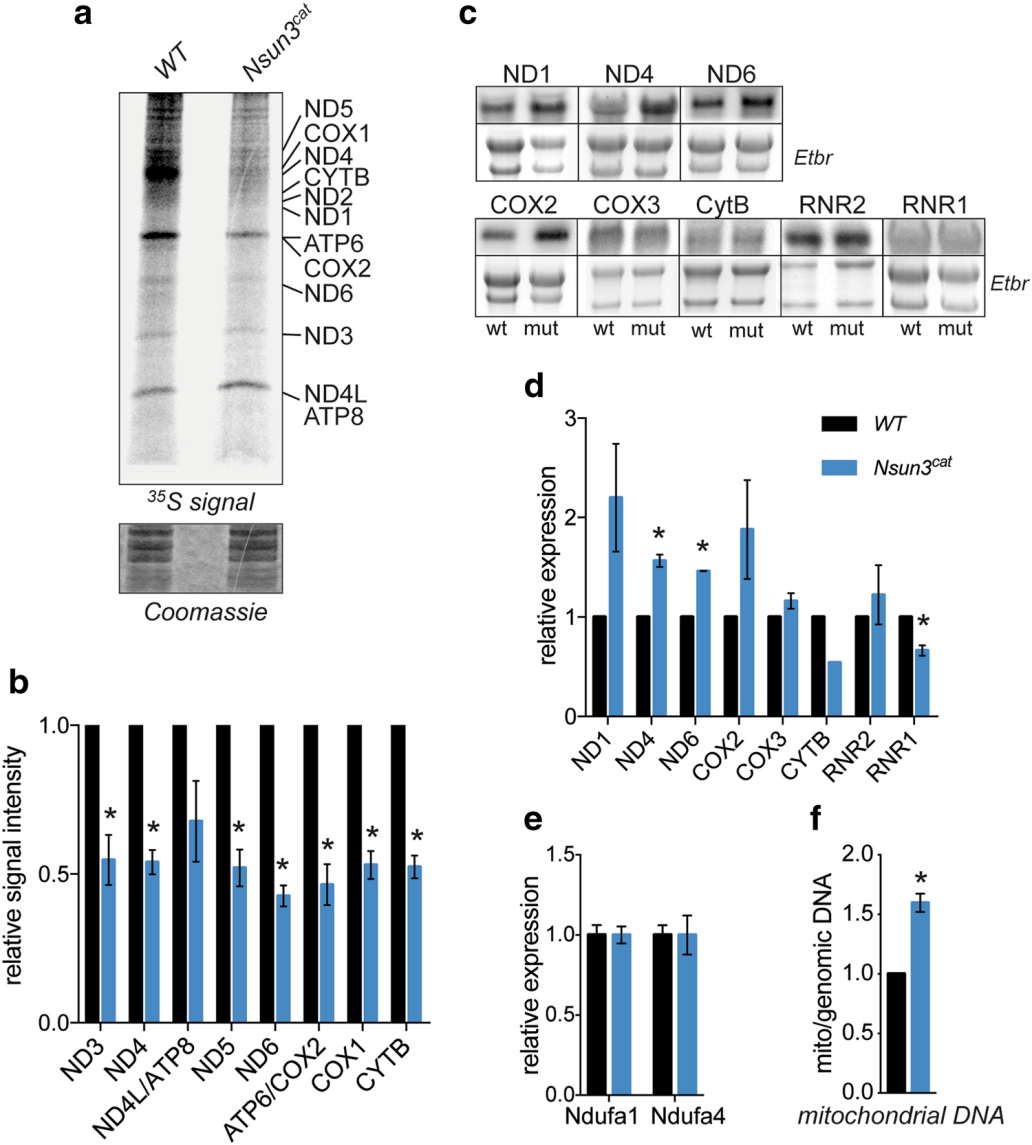
Inactivation of Nsun3 compromises mitochondrial protein translation and transcription. **a** Metabolic labelling experiments show reduced mitochondrial protein translation in *Nsun3* ^*cat/cat*^ ESCs. ^35^S-pulse-labelled mitochondrial proteins were fractionated on a 16% Tricine gel, stained with Coomassie brilliant blue (lower panel), dried, and exposed to phosphoscreen. **b** Quantification of radiation signals from **a** for the indicated mitochondrial proteins. Values represent mean ± SEM of three experiments. Statistical significance was calculated by multiple unpaired *t* test with Holm–Sidak correction (*p < 0.05). **c** Northern blot analysis of mitochondrial transcripts in wild-type and *Nsun3* ^*cat/cat*^ ESCs. Chemiluminescence signals of indicated transcripts (upper panels) and corresponding ethidium bromide (Etbr) stained 28S and 18S rRNA are shown. **d** Quantification of Northern blot signals shown in **c** normalized against rRNA band intensities. Values represent mean ± SEM of three experiments except for CytB where only one experiment was quantifiable. Statistical significance was calculated by multiple unpaired *t* test with Holm–Sidak correction (*p < 0.05). **e** Expression of nucleus-encoded *Ndufa1* and *Ndufa4* (electron-chain complex I components) transcripts was analyzed by RT-qPCR. Values represent mean ± SEM of three experiments. Statistical significance was calculated by unpaired *t* test (*p < 0.05). **f** Mitochondrial DNA content in *Nsun3* ^*cat/cat*^ ESCs was determined by qPCR of a mitochondrial gene normalized against a genomic gene and expressed relative to wild-type ESCs. Values represent mean ± SEM of three experiments. Statistical significance was calculated by unpaired *t* test (*p < 0.05)

Since deletion of human *NSUN3* was reported to compromise mitochondrial protein translation due to deficient methylation and formylation of C34 in mt-tRNA^Met^ [12–14], we tested if such a translation defect can also be detected in ESCs. To this end, we performed metabolic labelling experiments in wild-type and *Nsun3* ^*cat/cat*^ ESCs. Consistent with the earlier results in human cells, we found a pronounced decrease in mESC mitochondrial protein synthesis (Fig. 3a, b). By contrast, when we analyzed transcript levels of mitochondrially encoded genes using Northern blotting with strand-specific probes, we observed a trend towards higher mRNA amounts in *Nsun3* ^*cat/cat*^ cells. In particular, transcripts encoding complex I components ND1, ND4, and ND6 were more abundant in mutant ESCs, while changes in COX2, COX3, CYTB, and the mitoribosomal 16S rRNA (RNR2) did not reach significance, and 12S rRNA transcripts (RNR1) were reduced (Fig. 3c, d). We also analyzed the expression of two complex I components that are encoded in the nucleus using qPCR. In contrast to the mitochondrially encoded subunits, *Ndufa1* and *Ndufa4* transcript levels showed no difference between wild-type and *Nsun3*-mutant cells (Fig. 3e).

The elevated steady-state mitochondrial transcript levels in *Nsun3* ^*cat/cat*^ cells might originate from an increase in mitochondrial biogenesis in the mutant cells to compensate for the compromised protein translation. Indeed, when we measured mitochondrial DNA relative to nuclear DNA by real time PCR, we found a ~ 1.5-fold increase of mitochondrial DNA in *Nsun3* ^*cat/cat*^ ESCs compared to wild-type ESCs (Fig. 3f). The observed upregulation of mitochondrial transcripts and DNA content correspond well with the effects observed upon deletion of other factors that compromise mitochondrial translation (e.g. [34, 35]).

**Fig. 4:**
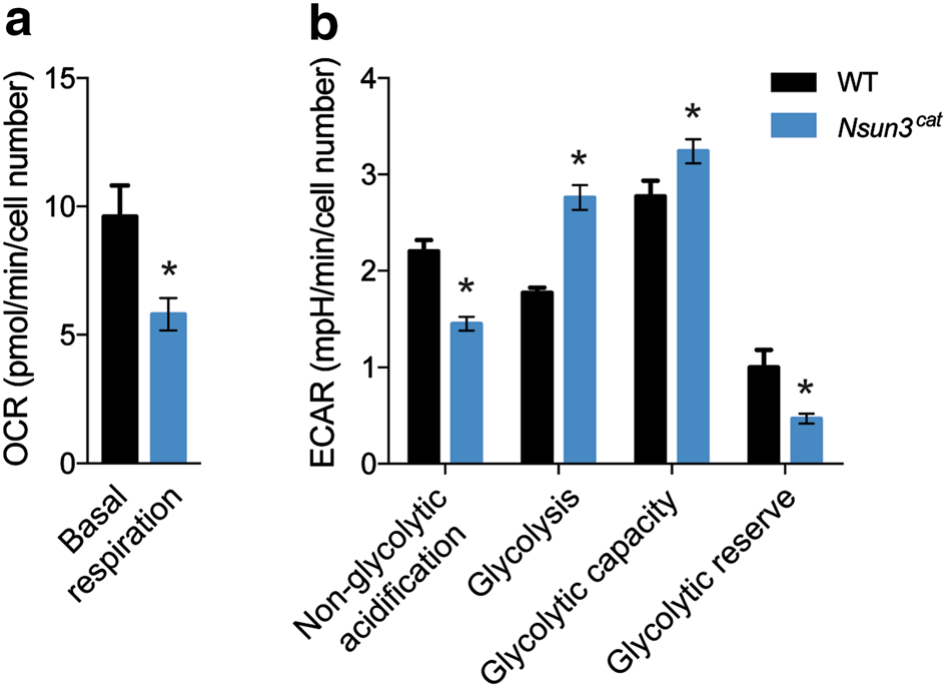
Mitochondrial respiration is decreased and glycolysis is increased in *Nsun3*-mutant ESCs. **a** Oxygen consumption rate (OCR) and **b** extracellular acidification rate (ECAR) were measured for wild-type and *Nsun3* ^*cat/cat*^ ESCs and statistical differences were determined by unpaired *t* test (*p < 0.05)

To study if decreased mitochondrial translation impacts on overall energy metabolism of *Nsun3* ^*cat/cat*^ cells, we measured oxygen consumption and found an almost 50% decrease in *Nsun3*-mutant versus wild-type cells (Fig. 4a). In contrast to other cell types, ESCs are less dependent on OXPHOS and obtain most of their energy from glycolysis [20, 36]. Measurement of extracellular acidification rates using a Seahorse Analyzer revealed a significantly increased basal glycolysis rate accompanied by decreased non-glycolytic acidification of *Nsun3* ^*cat/cat*^ versus wild-type ESCs. Addition of glucose led to increased acidification in the mutant compared to wild-type cells indicating higher glycolytic capacity, although the glycolytic reserve (i.e., the difference between basal and maximal glycolysis rate) was reduced (Fig. 4b). Thus, despite the fact that ESCs favour glycolysis over OXPHOS for ATP generation and that reduced electron transfer chain function in *Nsun3*-mutant cells further stimulates glycolysis, mutant cells are not able to reach cell densities that are comparable to wild-type cells (Fig. 2a).

### Nsun3 inactivation skews differentiation of ESCs towards mesendoderm

**Fig. 5:**
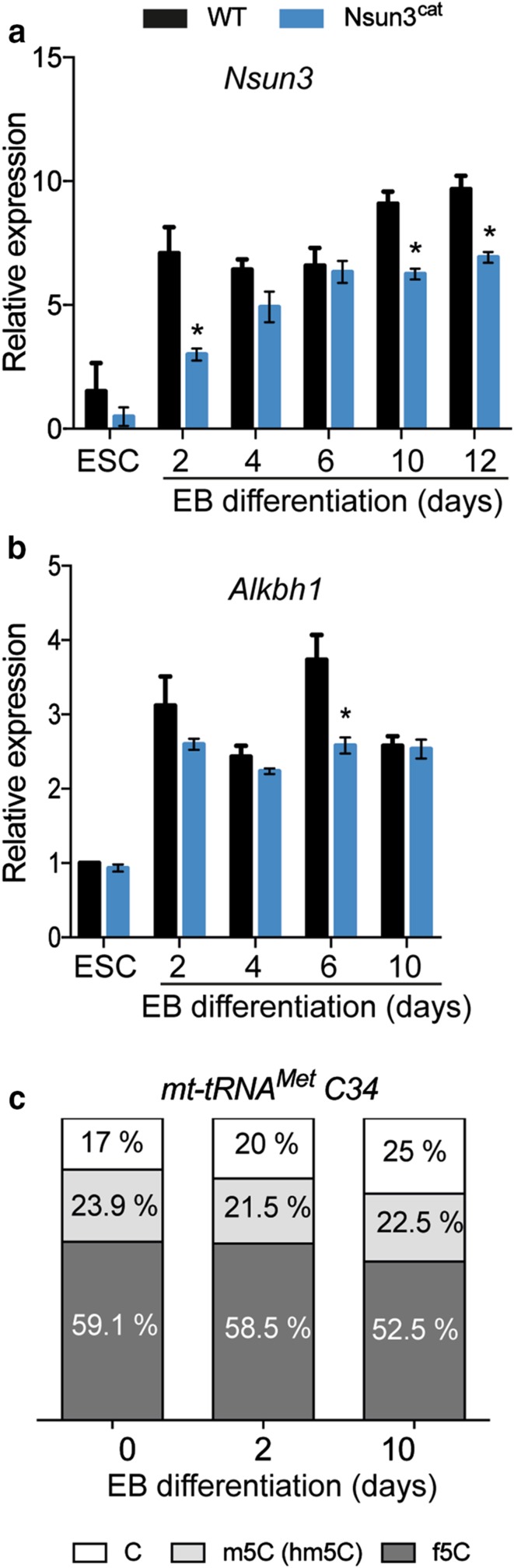
*Nsun3* and *Alkbh1* expression levels increase upon ESC differentiation. **a**, **b** RT-qPCR was performed on cDNA prepared from ESCs or embryoid bodies (EBs) of wild-type and *Nsun3* ^*cat/cat*^ cells at the indicated times of outgrowth on gelatine-coated plates. Transcript levels were normalized against *TATA*-*binding protein (TBP)* and are expressed relative to transcript levels in wild-type ESCs. **c** Modification state of mt-tRNA^Met^ C34 was analyzed in ESCs undergoing embryoid body differentiation using bisulfite and NaBH_4_-bisulfite sequencing as in Fig. 1c; the relative fractions of unmodified C, m5C, and f5C were calculated and plotted. For each time point, *n* = 20

Differentiation of ESCs is accompanied by a major metabolic transition from glycolysis-dominated to OXPHOS-based metabolism along with the stimulation of mitochondrial biogenesis [20]. To test the consequences of compromised mitochondrial protein translation for cell differentiation in *Nsun3*-mutant ESCs, we induced wild-type and *Nsun3* ^*cat/cat*^ cells to differentiate into embryoid bodies (EB) for 2 days using the hanging drop method followed by plating on gelatine-coated dishes for a period of 12 days and subsequent RT-qPCR analysis. We first determined *Nsun3* and *Alkbh1* expression levels in wild-type stem cells and at different stages of EB outgrowth. *Nsun3* is expressed in ESCs at a moderate level. Upon differentiation, expression rapidly increased during the first 2 days and afterwards continued to rise slightly until day 12 (Fig. 5a) suggesting enhanced need for Nsun3 during embryonic stem cell differentiation. Similar results were obtained for *Alkbh1,* although transcript increase was weaker than for *Nsun3* (Fig. 5b). RT-qPCR analysis of *Nsun3*-mutant cells revealed ~ 40% reduced *Nsun3* ^*cat*^ mRNA levels in ESCs and at day 2 of EB formation compared to wild-type cells. During days 4–6 of EB differentiation, *Nsun3* ^*ca*t^ mRNA increased but did not reach wild-type levels at later EB stages (Fig. 5a). The lower *Nsun3* ^*ca*t^ transcript levels may be caused by reduced mRNA stability due to the premature translational stop codon sensed by non-sense-mediated decay mechanisms [37]. By contrast, *Alkbh1* expression remained largely unaffected by *Nsun3*-mutation (Fig. 5b). Given the increase of *Nsun3* mRNA in ESCs undergoing differentiation, we also measured m5C34 and f5C34 of mt-tRNA^Met^ at days 2 and 10 of EB outgrowth (Fig. 5c). These experiments revealed largely unaltered C34 modification levels, suggesting that the increase in *Nsun3* and *Alkbh1* expression may be required to maintain a constant m5C:f5C ratio on C34 in the face of mitochondrial biogenesis during differentiation.

**Fig. 6:**
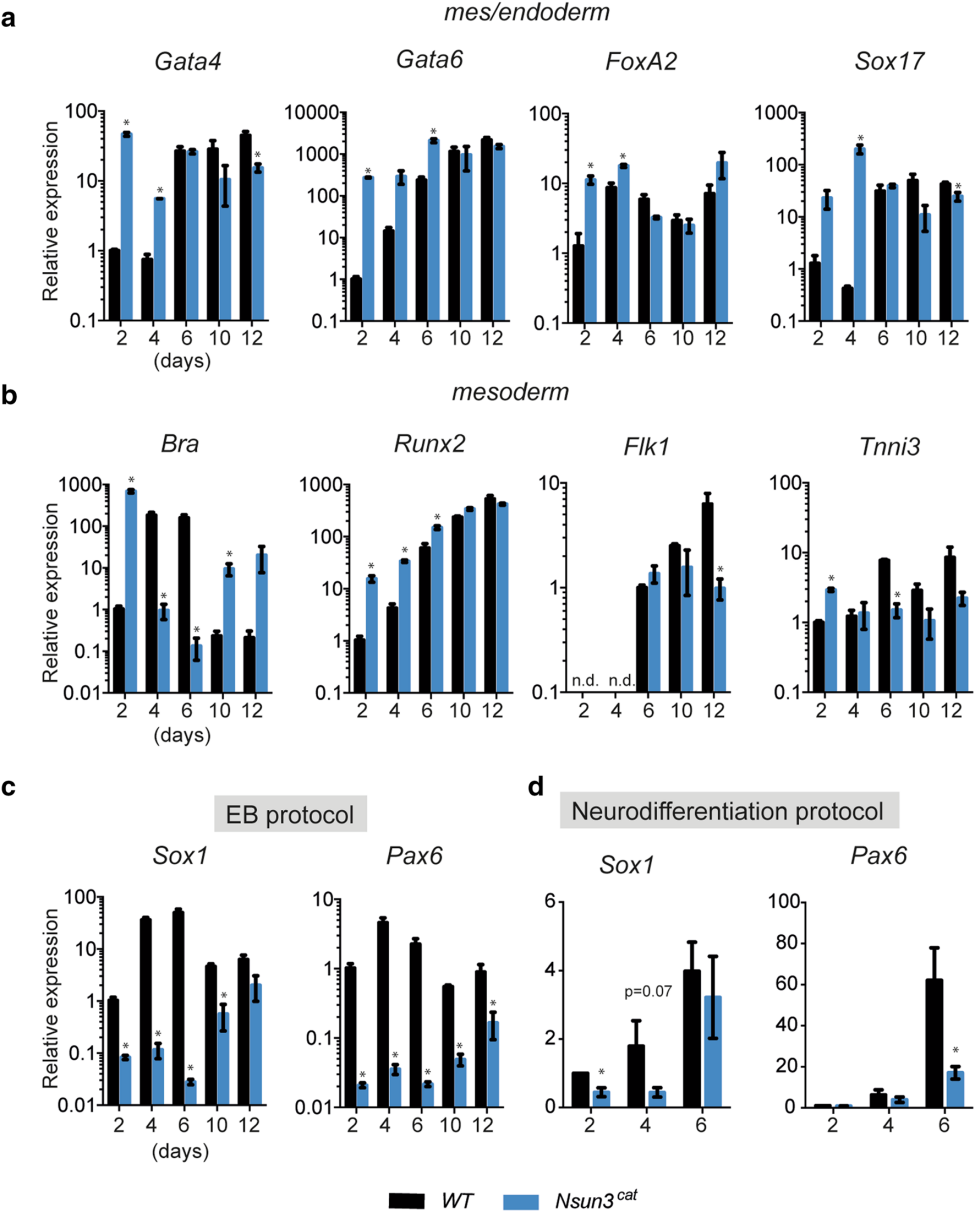
Neuroectoderm differentiation is severely impaired in *Nsun3* ^*cat/cat*^ EBs. **a**–**d** RT-qPCR was performed on cDNA prepared from embryoid bodies (EBs) of wild-type and *Nsun3* ^*cat/cat*^ cells at the indicated times of outgrowth on gelatine-coated plates (**a**–**c**) or from neural progenitor cells at the indicated times of differentiation (**d**). Expression of marker genes of **a** mes/endoderm, **b** mesoderm, and **c**, **d** neuroectoderm was tested. Transcript levels were normalized against *TATA*-*binding protein (TBP).* Values are relative to transcript levels in wild-type EBs at day 2. Mean values ± SEM of three experiments (performed on different days) are shown and statistical significance was calculated by multiple unpaired *t* test with Holm–Sidak correction (*p < 0.05). *n.d.* not detected

We then examined the differentiation potential of *Nsun3*-mutant ESCs. To this end, we analyzed the expression of various marker genes of the three germ layers and observed quite significant dysregulation (Fig. 6). In particular, the key regulators of the meso- and endodermal lineages (*Brachyury, FoxA2, Gata4* and *Gata6, Sox17, Runx2, Tnni3*) showed significantly increased expression in *Nsun3* ^*cat/cat*^ EBs particularly at the early stages of differentiation (Fig. 6a, b). Remarkably, *Brachyury* (*Bra*) transcript levels were significantly altered compared to wild-type cells throughout the EB outgrowth period. *Bra* exhibited a biphasic expression pattern in *Nsun3*-mutant cells with almost 1000-fold higher levels than in wild-type cells at day 2 of EB outgrowth followed by a sharp drop below wild-type levels at days 4-6, before it increased again significantly until day 12. These results suggest precocious upregulation of *Bra* in mutant versus wild-type cells. The observed strong early increase in endodermal markers *FoxA2, Gata4, Gata6,* and *Sox17* (Fig. 6a) and their subsequent return to wild-type levels or below at days 10–12 suggest that endodermal differentiation occurs earlier but not necessarily stronger in *Nsun3* ^*cat/cat*^ compared to wild-type EBs.

The strongest dysregulation, however, was observed for markers of the neuroectoderm. *Sox1* and *Pax6* showed dramatically lower levels in *Nsun3* ^*cat/cat*^ EBs at all stages but most pronounced at days 2–6 of outgrowth (Fig. 6c). Consistently, the pluripotency factor *Sox2*, which is also expressed in neuroectodermal cells [38], was significantly downregulated in *Nsun3*-mutant EBs at day 2 compared to wild type (adjusted *p* = 0.04; Supplementary Fig. S2). These results may indicate that inactivation of Nsun3 favours differentiation along the meso- and endodermal lineages at the expense of neuroectoderm differentiation programs. To further investigate this idea, we induced neurodifferentiation in *Nsun3* ^*cat/cat*^ and wild-type ESCs following published protocols [22]. Similar to the EB differentiation experiments, the expression of *Sox1* and *Pax6* was downregulated in *Nsun3* ^*cat/cat*^ cells compared to wild-type cells, supporting a delay in neuroectoderm differentiation of *Nsun3*-mutant cells (Fig. 6d). We conclude that ESC differentiation is affected by decreased mitochondrial activity caused by *Nsun3*-inactivation.

### *Nsun3* mutation affects Wnt signalling and production of mitochondrial ROS in ESCs

**Fig. 7:**
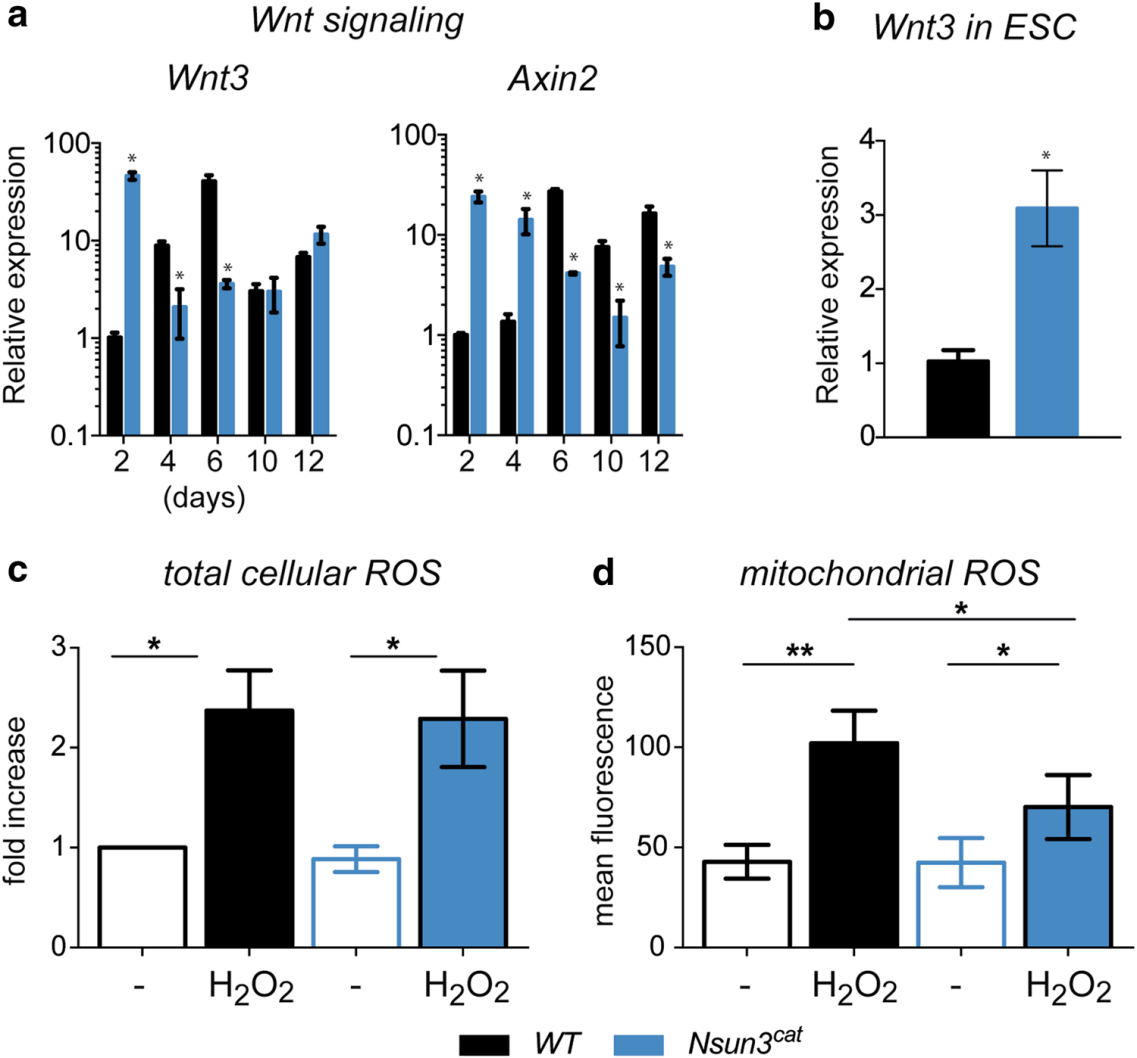
Nsun3 inactivation causes upregulation of Wnt signalling but does not affect basal ROS levels in ESCs. **a** RT-qPCR for *Wnt3* and *Axin2* was performed on cDNA prepared from EBs of wild-type and *Nsun3* ^*cat/cat*^ cells at the indicated times of outgrowth on gelatine-coated plates. **b** Expression of *Wnt3* is increased in *Nsun3*-mutant ESCs compared to wild-type cells. Normalization and quantification of PCR signals as in Fig. 6. **c** Quantification of total cellular ROS in control and H_2_O_2_-treated WT and *Nsun3* ^*cat/cat*^ cells by FACS revealed no differences between the cell lines. Relative mean ± SEM values of 5 experiments are shown. **d** Quantification of mitochondrial ROS by MitoTracker Red CM-H_2_XROS staining and fluorescence microscopy showed weaker ROS induction after H_2_O_2_ treatment in *Nsun3*-mutant versus wild-type cells. Mean ± SEM intensity values of eight experiments are shown. (*p < 0.05; **p < 0.01)

In search for potential causes of the observed strong overexpression of *Bra* during the early stages of EB formation in *Nsun3*-mutant cells, we examined if altered Wnt signalling might be responsible for this phenotype. In the mouse epiblast, Wnt3 signalling controls *Bra* expression [39, 40], and treatment of MEF cells with Wnt3 resulted in activation of *Bra* as well as *Wnt3* itself [41]. Consistent with these findings, we observed precipitate upregulation of *Wnt3* as well as of *Axin2* (another direct target of Wnt signalling) mRNA at day 2 of EB outgrowth in *Nsun3*-mutant EBs compared to wild-type cells. Therefore, it is likely that aberrant Wnt signalling controls the early upregulation of *Bra* and *Axin2* in differentiating ESCs leading to preferred differentiation into the mesendodermal lineage. Interestingly, it appears that the increase in *Bra* expression at later time points in *Nsun3* ^*cat/cat*^ but not in wild-type EBs may be independent of Wnt signalling, since neither *Axin2* nor *Wnt3* mRNA levels follow those of *Bra* (Fig. 7a).

Because *Wnt3* was overexpressed in *Nsun3* ^*cat/cat*^ cells at the earliest stages of differentiation, we examined *Wnt3* expression in ESCs. We found significantly elevated *Wnt3* mRNA levels already in *Nsun3* ^*cat/cat*^ compared to wild-type ES cells (Fig. 7b), suggesting that *Nsun3*-mutant ESCs are already primed towards the mes/endodermal lineage without losing pluripotency. To explain the connection between *Wnt3* upregulation and compromised mitochondrial translation caused by Nsun3 inactivation, we considered the possibility that the Wnt signalling pathway may be activated by aberrant levels of reactive oxygen species (ROS) in *Nsun3* ^*cat/cat*^ ESCs. The mitochondrial respiratory chain is a significant source of ROS and Wnt signalling is known to be sensitive to ROS [42]. Since reduced mitochondrial translation will affect the integrity of respiratory chain complexes and thus may lead either to increased or to decreased ROS production, we quantified total intracellular ROS levels in wild-type and *Nsun3*-mutant ESCs by FACS after 2′-7′-dichlorodihydrofluorescein diacetate (DCFH-DA) staining and mitochondrial ROS by fluorescence microscopy after MitoTracker Red CM-H_2_XROS staining. Surprisingly, we did not find any differences in basal ROS levels in wild-type versus *Nsun3* ^*cat/cat*^ ESCs (Fig. 7c, d). However, stressing the cells by treatment with H_2_O_2_ showed that mitochondrial ROS production in *Nsun3*-mutant ESCs was significantly weaker than in the wild type (Fig. 7d), whereas total cellular ROS induction was comparable in both cell lines (Fig. 7c).

Thus, while we cannot directly link the increased *Wnt3* levels in mutant ESCs to increased ROS levels, it is possible that the observed differentiation defects may, at least in part, involve the inability of the *Nsun3*-mutant cells to induce sufficient mitochondrial ROS to orchestrate the appropriate signalling cascades. To test this hypothesis, we measured mitochondrial ROS upon induction of differentiation by removal of LIF and 2i. However, we were unable to detect increased ROS levels either in wild-type or in *Nsun3*-mutant cells during the course of the experiment (Supplementary Fig. 3a), indicating that if ROS plays a role in differentiating ESCs, the required amounts are likely small or transient and beyond the detection limit of our method. Previously, it was reported that mESCs that exhibit low mitochondrial membrane potential and oxygen consumption preferentially differentiate into the mesodermal lineage [43]. To determine, if the observed precocious upregulation of mesodermal markers in *Nsun3*-mutant cells correlated with lower mitochondrial membrane potential in the mutant ESCs, we measured membrane potential in *Nsun3*-mutant and wild-type cells using TMRM (tetramethylrhodamine methyl ester) staining and FACS detection [43]. Overall, we found no differences in median TMRM fluorescence between the two cell lines (Supplementary Fig. 3b). H_2_O_2_ challenge reduced TMRM fluorescence significantly and more strongly in *Nsun3*-mutant than in wild-type cells indicating mitochondrial membrane damage, which is consistent with the data on impaired mitochondrial function shown in Fig. 3. When we looked at the fluorescence (and, therefore, membrane potential) distribution among both cell populations, we found a slightly higher number of cells in the lowest 5% of fluorescence intensity for *Nsun3*-mutant cells compared to wild-type cells (Supplementary Fig. S3b). This difference, however, was not significant, and, therefore, does not establish a clear correlation between membrane potential defects in *Nsun3*-mutant cells and their aberrant differentiation behavior.

## Discussion

The recent characterization of NSUN3 as a cytosine methyltransferase for mitochondrial tRNA^Met^ highlighted the importance of RNA modifications for mitochondrial function in human cells [12–14]. Mutations in the human *NSUN3* gene resulting in the expression of a severely truncated protein were associated with mitochondrial dysfunction and mitochondrial disease symptoms in a patient [13]. In this work, we show that catalytic inactivation of Nsun3 in mouse ESCs leads to reduced translation of mitochondrially encoded genes, upregulation of glycolysis and reduced oxygen consumption similar to the phenotypes observed in somatic human cells. Nsun3 activity, however, is also important for embryonic stem cell function and differentiation in the mouse despite the fact that ESCs unlike somatic cells rely mostly on glycolysis rather than oxidative phosphorylation for ATP generation. Nsun3 is the closest relative of Nsun4, which also localizes to mitochondria [44]. Nsun4 methylates C911 in mitochondrial 12S rRNA, yet in addition is required for the assembly of the large and small subunits of the mitoribosome in conjunction with the partner protein Mterf4 [35]. By analogy, it is a possibility that Nsun3, too, may have additional functions apart from its methyltransferase activity.

We also identified a cytosine methylation at position 47 of mitochondrial tRNA^Met^, which was not affected by *Nsun3*-inactivation. The methyltransferase Nsun2 is known to catalyze this modification at positions C48/49/50 in cytoplasmic tRNAs [45]. However, to date, Nsun2 is not known to localize to mitochondria [46], and it has not been detected in mitoproteome studies (http://www.mrc-mbu.cam.ac.uk/impi). Thus, the identity of the mitochondrial tRNA^Met^ C47 methyltransferase remains unknown at present. In cytoplasmic tRNAs, C48/49/50 methylation is thought to confer stability to the three-dimensional structure of the tRNA by participating in non-conventional base-pairing with nucleosides of the D-loop [47]. Moreover, cytosine methylation in the variable loop protects tRNAs from nucleolytic cleavage [48]. Whether this also occurs in mouse mt-tRNA^Met^ or why this nucleotide is not conserved in human mt-tRNA^Met^ remain open questions.

Although we found that *Nsun3* expression in self-renewing ESCs is low compared to differentiating stem cells, catalytical inactivation, which results in the absence of C34 methylation and formylation of mt-tRNA^Met^, significantly reduced mitochondrial translation and the proliferation rate of ESCs. This is consistent with previous results demonstrating that drug-mediated inhibition of mitochondrial function negatively affected ESC proliferation [49, 50], while increased respiration in mESCs can boost cell proliferation [51]. Similarly, compromising mitochondrial translation was found to inhibit proliferation of cultured and, therefore, mostly glycolytic MEF and HeLa cells [52–54]. The nature of this connection, however, remains unknown [55]. While it was shown in previous studies that treatment with drugs interfering with mitochondrial electron transfer and OXPHOS caused an upregulation of the key pluripotency factors *Oct4*, *Nanog,* and *Sox2* [49, 56], suggesting reinforcement of stem cell self-renewal, *Nsun3* inactivation did not affect the expression levels of these genes. This could be due to the fact that respiration is reduced but not abolished in *Nsun3*-mutant cells, and no increase in ROS levels is detected, while drug treatment by uncoupling electron transfer from ATP generation or by inhibiting complex III activity resulted in significant amounts of ROS [49, 50].

Our study suggests that Nsun3 is an important factor for ESC differentiation, in particular of neuroectoderm differentiation. Compromised neuronal differentiation was previously observed when mitochondrial activity was inhibited either by drug treatment or by mutations in electron transfer chain components [50, 57, 58]. However, in these cases, the defects in mitochondrial function were much more severe than in *Nsun3*-mutant cells, in which mitochondrial protein translation and oxygen consumption are only about 50% reduced. Moreover, while increased ROS levels accompanied reduced mitochondrial function in earlier studies [50], this was not the case in *Nsun3*-mutant ESCs. On the contrary, even upon oxidative stress induction, *Nsun3*-mutant cells were not able to produce the same amount of mitochondrial ROS as wild-type cells and their membranes were depolarized more severely. How the mitochondrial defects are connected to aberrant differentiation or to the altered *Wnt3* expression level in mutant ESCs remains to be addressed in future studies. The Nsun3^cat^ phenotype, however, attenuated respiratory function without abnormal ROS generation (which is often the case with drug-mediated inhibition of the electron transfer chain), makes this ES cell line a useful tool to study the complex interplay between mitochondrial activity and stem cell pluripotency and differentiation.

The impact of cytosine methylation of tRNAs on stem cell differentiation is not limited to Nsun3. Nsun2 also plays an important role in this process, as its mutation was shown to delay differentiation of skin and testis in mice [59, 60]. Methylation of tRNAs by Nsun2 protects from nucleolytic cleavage [48, 61] and serves to regulate protein translation rates, which appears to be involved in controlling stem cell identity [45]. Thus, cytosine methylation of tRNAs regulating decoding potential or stability of tRNAs is emerging as a critical mechanism in the governing of stem cell fate and function.

## Electronic supplementary material

Below is the link to the electronic supplementary material.

Supplementary material 1 (PDF 323 kb)
