# Supplementary material for: RNA cytosine methyltransferase Nsun3 regulates embryonic stem cell differentiation by promoting mitochondrial activity
Source: Cell Mol Life Sci. 2017 Nov 4;75(8):1483–97. doi: 10.1007/s00018-017-2700-0 (PMC5852174; doi:10.1007/s00018-017-2700-0)
Supplement: Supplementary file 1 — Supplementary material 1 (PDF 323 kb) [file 18_2017_2700_MOESM1_ESM.pdf]

# Supplementary Figures

## RNA cytosine methyltransferase Nsun3 regulates embryonic stem cell differentiation by promoting mitochondrial activity

Lukas Trixl[1§], Thomas Amort[1§], Alexandra Wille[1§], Manuela Zinni[1], Susanne Ebner[2], Clara Hechenberger[1], Felix Eichin[1], Hanna Gabriel[1], Ines Schoberleitner[1], Anming Huang[1], Paolo Piatti[3], Roxana Nat[4], Jakob Troppmair[2], Alexandra Lusser[1*]

[1] Division of Molecular Biology, Biocenter, Medical University of Innsbruck, 6020 Innsbruck, Austria

[2] Daniel Swarovski Research Laboratory, Department of Visceral, Transplant, and Thoracic Surgery, Medical University of Innsbruck, 6020 Innsbruck, Austria

[3] Zymo Research Corp., Irvine, CA, USA

[4] Institute for Neuroscience, Medical University of Innsbruck, 6020 Innsbruck, Austria.

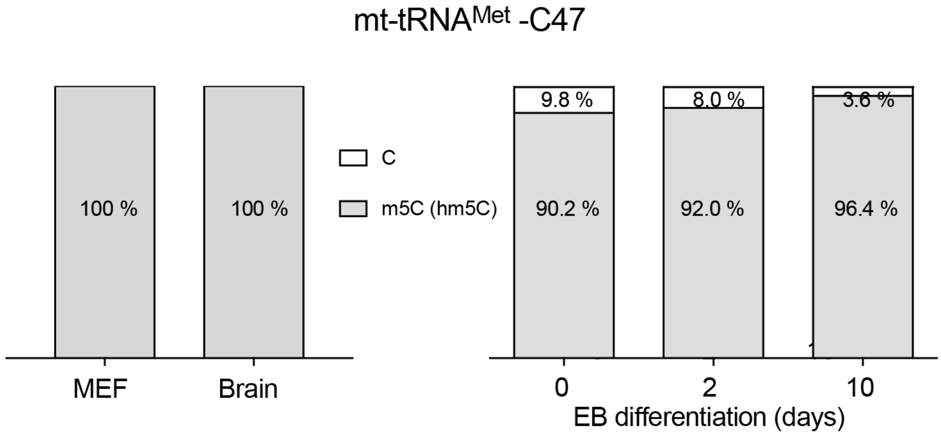


**Supplementary Figure S1**. C47 of mt-tRNA$^{Met}$ shows complete methylation in mouse embryo fibroblasts (MEF) and in whole brain from adult mice, and near complete methylation in differentiating embryoid bodies (EBs). RNA was analyzed by bisulfite sequencing (n=10 for MEF, brain; n=20 for EB)

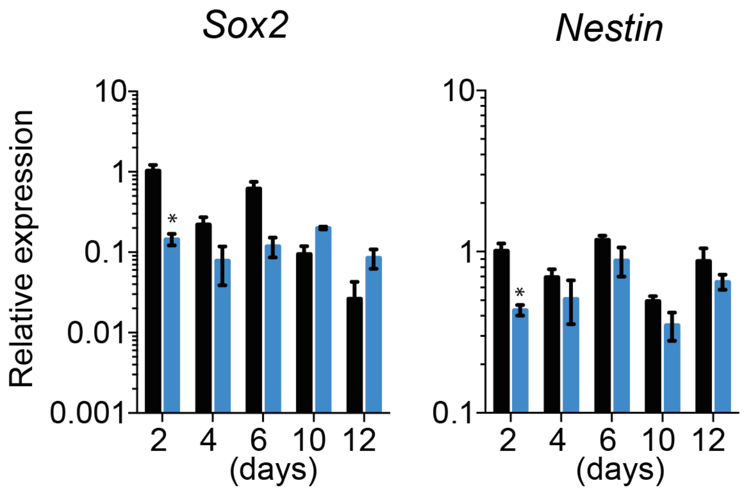


**Supplementary Figure S2** Neuroectoderm differentiation is impaired in *Nsun3*$^{cat/cat}$ EBs. RT-qPCR was performed on cDNA prepared from embryoid bodies (EBs) of wild type and *Nsun3*$^{cat/cat}$ cells at the indicated times of outgrowth on gelatine-coated plates. Expression of neuroectoderm marker genes *Sox2* and *Nestin* was tested. Transcript levels were normalized against *TATA-binding protein (TBP).* Values are relative to transcript levels in wild-type EBs at day 2. Mean values ± SEM of three experiments (performed on different days) are shown and statistical significance was calculated by multiple unpaired-t-test with Holm-Sidak correction (*p<0.05)

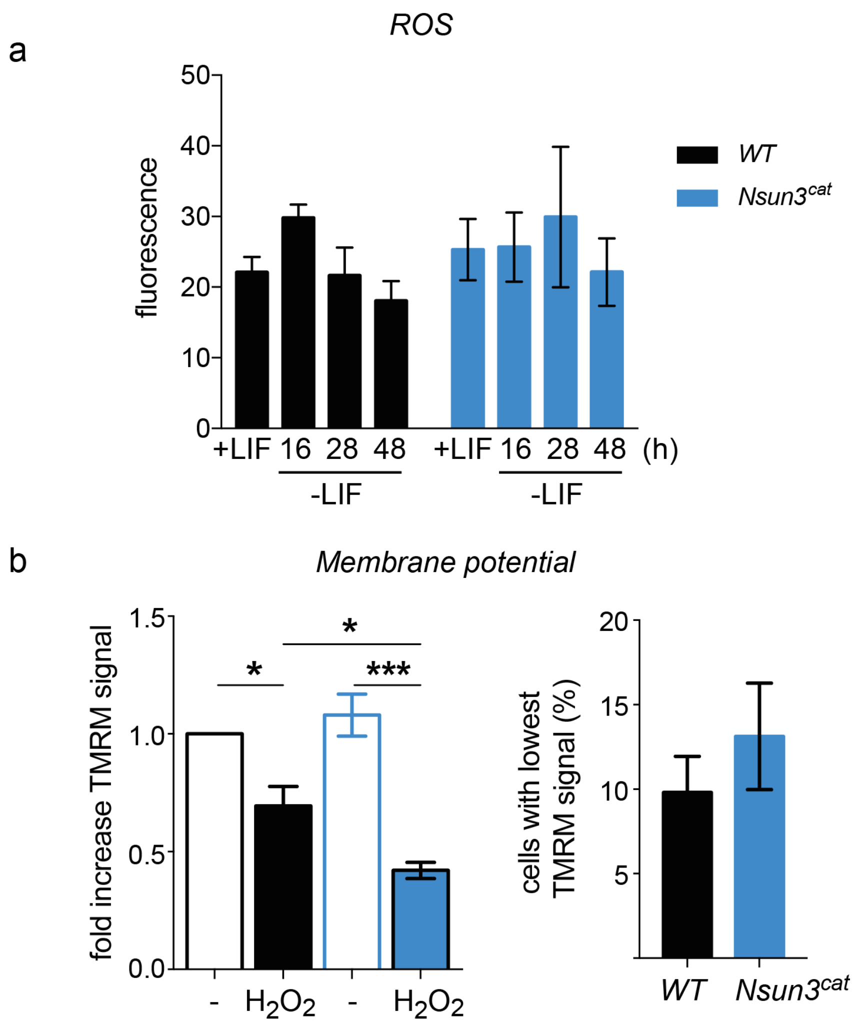


**Supplementary Figure S3**. **a** ROS levels are similar in wild-type (WT) and *Nsun3*$^{cat/cat}$ cells upon LIF and 2i removal. Mitochondrial ROS was quantified by MitoTracker Red CM-$H_2$XROS staining and fluorescence microscopy at the indicated times after LIF+2i removal. Mean fluorescence values ±SEM of four or five independent experiments are shown. No statistically significant ($p<0.05$) differences were found by unpaired t-test. **b** Mitochondrial membrane potential was assessed by staining cells with TMRM and quantification by FACS. Wild-type and mutant ESCs were either untreated or treated with 1 mM $H_2O_2$ for 30 min before TMRM measurement. Geometric mean values ±SEM of five independent experiments normalized against the geometric mean of untreated wild-type ESCs are shown in the left plot. For the plot on the right, numbers of cells within the bottom 5% of fluorescence intensity were determined and expressed relative to the total number of cells scanned (total number of cells per experiment ~4,800; 5 experiments). Statistical significance was calculated by one-way ANOVA (*$p<0.05$; ***$p<0.001$).
